# HSCT-GAVE as a Manifestation of Chronic Graft versus Host Disease: A Case Report and Review of the Existing Literature

**DOI:** 10.1155/2018/2376483

**Published:** 2018-03-12

**Authors:** Michael J. Grant, Mitchell E. Horwitz

**Affiliations:** Adult Blood and Marrow Transplant Program, Duke University Medical Center, Durham, NC, USA

## Abstract

Gastric antral vascular ectasia or “watermelon stomach” is a significant cause of nonvariceal upper GI bleeding and is characterized by red, tortuous ectatic vessels along longitudinal folds in the gastric antrum. The existing literature links GAVE to patients with cirrhosis, scleroderma, bone marrow transplantation, and chronic renal failure among other associations, but its pathophysiology remains ill-defined. Over 30 cases of hematopoietic stem cell transplant-related GAVE (HSCT-GAVE) have been reported in the literature to date and there are likely many more that go undiagnosed or are attributed to another cause of upper gastrointestinal bleeding. Interestingly, a busulfan-containing conditioning regimen has been the primary factor implicated in the etiology of HSCT-GAVE because this was common to all cases in the literature to date. Here, we present the first case of HSCT-GAVE in a patient that was treated with a non-busulfan-containing conditioning regimen. We propose a link between chronic GVHD and the development of HSCT-GAVE that is supported by a similar development of GAVE in patients with systemic sclerosis.

## 1. Introduction

Despite being first described in 1953, gastric antral vascular ectasia (GAVE) was more clearly defined in 1984 by Jabbari et al. [1, 2]. Most patients with this clinical entity present with either occult bleeding causing transfusion dependent iron deficiency anemia or severe acute upper GI bleeding [3]. The condition is diagnosed endoscopically and is characterized by visible columns of red, tortuous, ectatic vessels along the longitudinal folds of the gastric antrum. This endoscopic appearance is pathognomonic for GAVE [4]. Histologically, GAVE consists of vascular ectasia within the mucosa as well as fibrin thrombi, fibrohyalinosis, and spindle cell proliferation [4, 5]. These features are also pathognomonic for GAVE [5]. GAVE accounts for 4% of all nonvariceal upper GI bleeding cases [3]. Cirrhosis is found in 30% of all patients with GAVE, but it has also been associated with scleroderma, bone marrow transplantation, chronic renal failure, renal transplantation, ischemic heart disease, valvular heart disease, familial Mediterranean fever, and acute myelogenous leukemia [3].

There have been close to 35 cases of hematopoietic stem cell transplant-related GAVE (HSCT-GAVE) described in the literature. This association was first described in 1994 by Marmaduke et al. who retrospectively identified 10 patients with gastric vascular ectasia after undergoing bone marrow transplantation [6]. Due to the severity of bleeding in most of the reported cases, it is important to consider this clinical entity in patients that are in the post-stem cell transplant setting who develop hematemesis, melena, or new onset anemia. This is especially relevant because it can be responsive to both medical and endoscope-assisted therapeutic interventions. However, the etiology of HSCT-GAVE remains unclear. Its relative clinical rarity makes elucidating a pathophysiological mechanism both difficult and inherently imperfect. Hence, authors have proposed mechanisms based only on case similarities in the existing literature. A unifying mechanism may help us more confidently and reliably treat patients with this disease entity which is shown to be associated with significant morbidity and mortality. So far, authors have implicated conditioning regimen toxicity, portal hypertension from venoocclusive disease of the liver, thrombotic microangiopathy, and chronic graft versus host disease (GVHD). A busulfan-containing conditioning regimen has been common to all cases of HSCT-GAVE and has been the primary factor implicated in the etiology due to its ubiquity in the existing literature. Here, we present the first case of HSCT-GAVE in a patient that was treated with a non-busulfan-containing conditioning regimen. We argue that it is a form of GVHD instead of a sequela of the transplantation conditioning regimen.

## 2. Case

**Figure 1:**
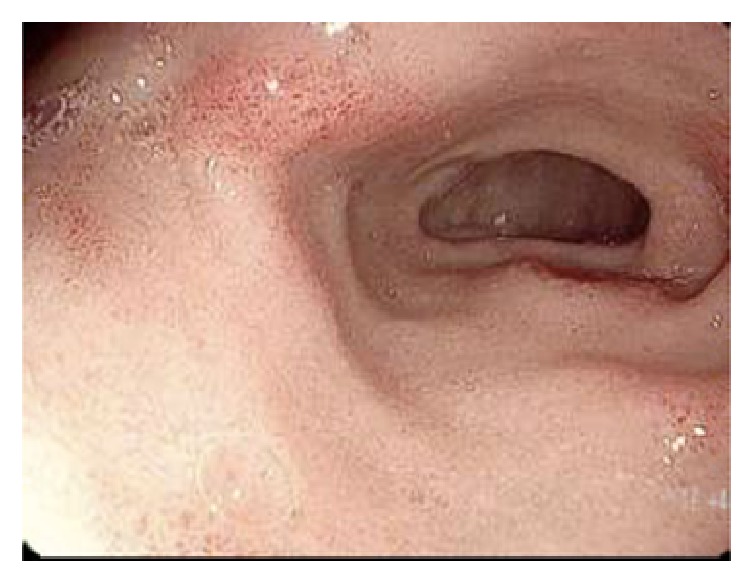
Endoscopic appearance of duodenal inflammation consistent with chronic GVHD on biopsy. This patient also had similar lesions in the gastric antrum to that depicted in this figure.

Our patient is a 46-year-old man with Philadelphia chromosome positive acute lymphoblastic leukemia. He received induction therapy with cyclophosphamide, vincristine, adriamycin, and dexamethasone, alternating with methotrexate and cytarabine (HyperCVAD). He also received dasatinib for 6 cycles along with intrathecal methotrexate. Follow-up bone marrow biopsy and aspirate demonstrated a complete remission with negative BCR/ABL by FISH. He was referred for a hematopoietic stem cell transplantation and received myeloablative conditioning with 1350 cGy of total body irradiation as well as cyclophosphamide 120 mg/kg. This was followed by a mobilized peripheral blood stem cell transplant from an HLA 8/8 matched unrelated donor (MUD) on day 0. He received GVHD prophylaxis in the form of tacrolimus and methotrexate. Bone marrow biopsy and aspirate on day +30 showed normocellular bone marrow (30%) with trilineage hematopoiesis and were negative for increased blasts. On day +72, he had an upper endoscopy for epigastric pain associated with nausea and vomiting and was found to have patchy granular gastric mucosa as well as patchy duodenitis (Figure 1). Biopsies of the gastric antrum and duodenum revealed increased apoptotic cells and were consistent with acute gastric and duodenal GVHD. IHC staining was negative for CMV. The patient was treated with prednisone and symptoms resolved.

On day +270 after discontinuing tacrolimus in preparation for a dental procedure, he developed an extensive morbilliform eruption on his head, neck, trunk, and extremities, as well as elevated transaminases consistent with chronic GVHD of the skin and liver. He was placed on a prednisone taper along with rapamycin with improvement in liver enzymes and skin rash. He continued to do well with regular follow-up with his oncologist.

**Figure 2:**
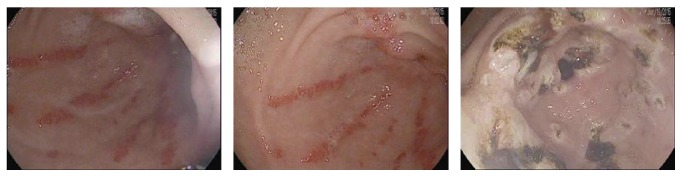
Gastric antral vascular ectasia seen in our patient on endoscopic examination 864 days following transplantation.

On day +744, he reported progressive shortness of breath with exertion as well as dark stools which precipitated a visit to his primary care physician. Labs were significant for a hemoglobin of 8.1 g/dl, down from a baseline of 12 g/dl. He was also found to be iron-deficient at that time. He received 2 units of packed red blood cells and was referred for upper and lower endoscopies to rule out GI bleed. Upper endoscopy revealed “watermelon stomach” consistent with a diagnosis of GAVE and one actively bleeding vessel was identified and cauterized. He also had one treatment of Argon Plasma Coagulation (APC) at the time of diagnosis. A colonoscopy and video capsule endoscopy to evaluate the small bowel did not reveal a potential source of bleeding. Thus, GAVE was thought to be the primary source of his symptoms and blood loss. Dark stools and crampy abdominal pain persisted and he required greater than 10 units of blood over the ensuing four months. Repeat endoscopy at our institution on day +864 redemonstrated GAVE (Figure 2) and APC was again used to treat lesions in the gastric antrum. Prednisone was increased from 5 mg daily to 10 mg daily due to a suspicion that this may be related to GVHD. His hemoglobin recovered to 11-12 g/dl and his symptoms resolved after this second treatment. Upper endoscopy on day +901 showed complete resolution of GAVE and a normal appearing mucosa in the gastric antrum. In the posttransplant period, immediate or long-term, the patient had no evidence of sinusoidal obstructive syndrome or thrombotic microangiopathy. Aside from the acute gastric GVHD and mild transaminase elevation consistent with liver GVHD discussed above, the course was complicated only by ongoing and difficult-to-control diffuse chronic sclerodermatous GVHD of the skin requiring several courses of immunosuppressive therapy. Skin biopsy of one of his thickened skin plaques revealed enlargement of the dermal collagen fibers consistent with sclerosis.

## 3. Discussion

### 3.1. Review of the Literature

The association between GAVE and HSCT patients was first brought to light in 1994 by Marmaduke et al. who identified 10 patients with gastric vascular ectasia after bone marrow transplantation [6]. All 10 had received conditioning with busulfan and cyclophosphamide without total body irradiation and the authors proposed that conditioning agent toxicity was likely implicated in the pathogenesis. GAVE was diagnosed in these patients on average 166 days following transplantation, and the patient with the longest time between transplant and discovery of GAVE was diagnosed on day +475 [6]. In 1996, Tobin et al. published a case series of 6 male patients who developed GAVE in the posttransplant period [7]. Factors that were common to all patients besides male sex were busulfan-cyclophosphamide therapy as well as VOD (venoocclusive disease) of the liver [7]. In 2003, Ohashi et al. identified 5 patients with HSCT-GAVE presenting a median of 95 days after transplantation (range: 65–208 days) [8]. All patients were conditioned with busulfan and showed evidence of thrombotic microangiopathy (TMA), but only one patient had hepatic venoocclusive disease. Ohashi et al. proposed that the anatomic distribution of the GAVE lesions alluded to a direct mucosal damage effect from oral administration of high-dose busulfan. The use of metoprolol to successfully treat three patients with bleeding from GAVE led authors to postulate that GAVE in this setting may be associated with portal hypertension. Several other cases have been reported, all of which included an oral busulfan-containing conditioning regimen [9–11]. In 2013, Fukuda et al. presented 2 cases of HSCT-GAVE developed after IV busulfan-containing regimens (IV Bu/cyclophosphamide and IV Bu/cytosine arabinoside) [12]. These were the first two cases of HSCT-GAVE after a regimen that did not contain PO busulfan. Though there could be no direct gastric antral mucosal contact in these cases, the authors stated that busulfan might still be implicated in the pathogenesis. Instead of direct contact to the mucosal membrane, they implicated “increased portal vein pressure caused by sinusoidal obstruction under the influence of busulfan, [which] induces ectasia of antral vein” [12]. Neither patient had signs of sinusoidal obstruction. Most recently, Hirayama et al. described a 6-year-old boy with AML who was conditioned with oral busulfan, cytosine arabinoside, and cyclophosphamide before receiving a stem cell transplant from an HLA-matched sibling donor [13]. Dry mouth symptoms led to a lip biopsy, which confirmed chronic GVHD on day 90 after cyclosporin was discontinued. On day +120, he developed epigastralgia followed by hematemesis and a diagnostic endoscopy and subsequent biopsy demonstrated GAVE. Immunohistological staining revealed CD4+ T-cells, CD8+ T-cells, and CD68+ monocytes/macrophages and the absence of B-cell and NK cell infiltrates histologically consistent with descriptions of chronic GVHD lesions. The authors proposed that this and the fact that the GAVE lesions responded to oral corticosteroid therapy suggest that HSCT-GAVE may be linked to GVHD. Follow-up endoscopy one month after prednisolone revealed significant improvement of GAVE. Interestingly, this patient also had evidence of a protein losing enteropathy, a condition which has also been described in association with autoimmune disease and chronic GVHD.

### 3.2. Pathophysiology

There have been several proposed mechanisms for HSCT-GAVE. One theory is that it is mediated by portal hypertension related to the development of venoocclusive disease of the liver in patients after bone marrow transplantation. This is based on the fact that GAVE is seen in nontransplant patients with cirrhosis and portal hypertension. However, studies in the nontransplant population have reported two separate endoscopic appearances of GAVE with different epidemiological aspects: the diffuse appearance of GAVE in patients with cirrhosis and the typical “watermelon stomach” appearance of GAVE in noncirrhotic patients [3, 14, 15]. Moreover, many of the patients with HSCT-GAVE did not have any evidence of sinusoidal obstruction syndrome or venoocclusive disease of the liver (VOD), which manifests as some combination of elevated bilirubin, ascites, right upper quadrant abdominal pain, and sudden weight gain. Furthermore, in cirrhotic patients with GAVE, portal decompression with transhepatic portosystemic shunt (TIPS) has been shown not to be a successful intervention, suggesting against a mechanism that has to do with portal hypertension leading to GAVE [16, 17].

GAVE has been well described in patients with systemic sclerosis/scleroderma with an estimated prevalence of 5.7% in this population [18]. In fact, some studies suggest that this number may be substantially higher considering asymptomatic patients with endoscopic findings of GAVE. We know that the clinical manifestations of chronic GVHD are similar to autoimmune collagen vascular diseases such as scleroderma, and a pathophysiological link between autoimmune conditions and chronic GVHD may be present. Autoantibodies are an expression of B-cell hyperactivity promoted by autoreactive T-cells in autoimmune diseases and by donor T-cells in chronic GVHD [19]. Autoantibodies may cross-react with specific proteins in the gastric mucosa and submucosa leading to inflammation, vascular endothelial cell injury, and ultimately the local release of factors resulting in thrombus formation in these small vessels [19, 20]. Additionally, GAVE in scleroderma patients (SSc-GAVE) has been associated with specific antibodies. One study demonstrated that patients with SSc-GAVE were positive for anti-RNA polymerase III at a rate of 50% compared to 16% in patients with scleroderma and no evidence of GAVE [20, 21]. GAVE lesions in scleroderma have been reported to resolve with immunosuppressive therapy, and gastric biopsies from cases of SSc-GAVE also show CD4+ T-cell infiltration [19, 22, 23]. These features point to SSc-GAVE being an immune-mediated process. Similarly, there is evidence of CD4+ T-cells, CD8+ T-cells, and CD68+ monocytes infiltrates in histopathological specimens of the HSCT-GAVE lesions [13]. The connection between scleroderma and chronic GVHD (cGVHD) has been described in depth before and the overlap of GAVE lesions in both of these conditions seems likely to be related to a common immune-mediated mechanism. Microchimerism, caused by class II HLA compatibility, may be involved in the pathogenesis of scleroderma [24]. This is one of the parallels between chronic GVHD and scleroderma. In fact, it has been suggested before that scleroderma may be a form of chronic GVHD and there is growing evidence to support this [24–27]. In summary, there seems to be an immune-mediated link between chronic GVHD and the development of HSCT-GAVE that is supported by a similar development of GAVE in patients with systemic sclerosis.

Our case is the first in the existing literature that describes a patient after stem cell transplantation who developed GAVE in the absence of a busulfan-containing conditioning regimen. Busulfan has been common to all cases of HSCT-GAVE and has been the primary factor implicated in the etiology of HSCT-GAVE. Previous theories on HSCT-GAVE center around busulfan administration and subsequent mucosal damage, endothelial injury, and the resulting portal hypertension [8, 28]. However, our patient received conditioning with cyclophosphamide and total body irradiation and had occurrence of GAVE over two years after his transplant. Though some cases have been reported in the early posttransplant period, there are several cases like ours where GAVE was reported after a significant interval of time from the conditioning regimen, calling into question this relationship [4, 28]. Only 3 cases have been reported within 30 days of transplantation [7, 28]. HSCT-GAVE was thought to have a connection to direct gastric mucosal contact with busulfan and the development of vascular ectasia from this exposure. However, the report of two patients who developed GAVE after HSCT with IV busulfan as part of their conditioning regimen shows that direct mucosal contact is not necessary [12]. Additionally, Hirayama described the cooccurrence of GAVE and protein losing enteropathy against a background of chronic GVHD [13]. In this case, GAVE lesions resolved after the patient was treated with cyclosporin and prednisolone. As discussed above, the authors noted histopathological parallels between HSCT-GAVE and chronic GVHD. Our patient also had evidence of GVHD in the duodenal bulb and gastric antrum after transplantation and chronic GVHD in the form of sclerodermatous skin changes and hepatitis. Since our patient did not have exposure to a busulfan-containing conditioning regimen, we hypothesize that HSCT-GAVE is a manifestation of chronic GVHD and not a sequela of the conditioning regimen as was previously proposed.

## 4. Conclusions

HSCT-GAVE is a well-reported entity, the pathophysiology of which has yet to be elucidated. The coexistence of documented gastrointestinal and severe sclerodermatous cutaneous chronic GVHD in our patient leads us to believe that HSCT-GAVE is another manifestation of chronic GVHD. We believe this is supported by the similarities between chronic GVHD and autoimmune conditions such as scleroderma and the propensity for patients to develop GAVE in association with both HSCT and scleroderma. There is likely an immune-mediated pathophysiological mechanism that is related to chronic GVHD in patients with HSCT-GAVE. Considering our proposed pathophysiological link between HSCT-GAVE and chronic GVHD, it is possible that this condition would respond to immunosuppressive therapy. Our case along with many of the other cases described above showed resolution with a number of different endoscopic electrocoagulation modalities.
